# Association of the uric acid to high-density lipoprotein cholesterol ratio with breast cancer odds: a retrospective study

**DOI:** 10.3389/fmed.2026.1741976

**Published:** 2026-01-29

**Authors:** Qingyu Ren, Guoqing Li, Eryu Liu, Jiaqin Zhou, Ling Chen, Xiang Gao

**Affiliations:** 1 Department of Oncology, Affiliated Hospital of Jiangnan University, Wuxi, China; 2 Department of Breast and Thyroid Surgery, Affiliated Hospital of Jiangnan University, Wuxi, China

**Keywords:** breast cancer, high-density lipoprotein cholesterol, metabolic biomarker, odds prediction, UHR, uric acid

## Abstract

**Objective:**

This study looks into the relationship between serum uric acid levels and the ratio of serum uric acid to high-density lipoprotein cholesterol (UHR) with the likelihood of breast cancer in women. It also evaluates its value as a biomarker related to metabolism for breast cancer.

**Methods:**

This retrospective study included 500 women (279 breast cancer patients and 221 with benign breast nodules). We calculated the uric acid to high-density lipoprotein cholesterol ratio (UHR) using preoperative lab data. Multivariable logistic regression analysis analyzed its relationship with the likelihood of getting breast cancer. Meanwhile, ROC curves and restricted cubic spline analyses assessed its diagnostic effectiveness and dose–response relationship.

**Results:**

In the breast cancer group, UHR was significantly higher than that of the benign group (0.10 ± 0.04 vs. 0.09 ± 0.03, *p* < 0.001). Multivariate analysis showed that for every 100-unit increase in UHR, the odds of developing breast cancer increased by 9 to 13%, and subjects with UHR ≥ 0.091 had a 63% increased odds of developing breast cancer (OR = 1.63, 95% CI: 1.03–2.59). However, UHR showed moderate diagnostic effectiveness (AUC = 0.62), with a sensitivity rate of 80%. Additionally, analysis using restricted cubic splines confirmed a positive linear correlation in a dose–response manner, indicating there was no threshold effect.

**Conclusion:**

Research has found that UHR (ultra-high risk) is linked to a higher chance of developing breast cancer. This suggests that UHR could help predict breast cancer risk. In the future, we need to confirm our findings with a bigger group of people and keep digging into the reasons behind this.

## Introduction

Globally, breast cancer still tops the list of malignancies in women, and its onset stems from a tangled interplay of inherited odds, shifting hormones and the thousand tiny choices that make up daily life (1, 2). In recent years, the association between metabolic disorders and tumor development has become a hot topic in medical research. Among them, the roles of lipid metabolism abnormalities and uric acid metabolism imbalance in the pathological process of breast cancer have gradually received attention (3, 4), however, research on their combined effects remains scarce.

Lipid metabolic reprogramming is one of the core characteristics of tumor cells. High-density lipoprotein cholesterol (HDL-C), often referred to as the “protective cholesterol,” is closely associated with the odds of various cancers when its levels are abnormal (5, 6). A study indicated that regardless of population heterogeneity or how obesity and metabolic status are defined, patients with metabolically healthy obesity (MHO) have a lower cancer odds than those with metabolically unhealthy obesity (MUO) (7). In patients with breast cancer, the level of high-density lipoprotein cholesterol (HDL-C) decreases significantly after chemotherapy. This change is associated with metabolism-related factors such as the patient’s age and body mass index (BMI), which further reflects the close connection between HDL-C metabolism and the disease course of breast cancer (8, 9).

Uric acid is the end product of purine metabolism, and the association between its metabolic abnormalities and chronic inflammation, oxidative stress, as well as the regulation of cell proliferation has been widely confirmed (10, 11). Clinical studies have demonstrated that hyperuricemia may reduce patients’ survival rates by promoting the proliferation and metastasis of breast cancer cells. Meanwhile, uric acid levels exhibit a unique J-shaped association with breast cancer odds, suggesting the dual regulatory potential of disturbed uric acid homeostasis in the pathogenesis of breast cancer (12). Notably, uric acid metabolism and lipid metabolism are not independent processes. Previous studies have observed a significant non-linear association between lipid ratios and hyperuricemia in cancer patients, confirming the close crosstalk between the two in the metabolic network.

Although the individual effects of uric acid and HDL-C have been initially explored, the association between the uric acid to high-density lipoprotein ratio (UA/HDL)—a key indicator integrating their metabolic status—and breast cancer odds remains unclear. Most existing studies have focused on single indicators or other lipid ratios, with a lack of systematic analysis on the UA/HDL ratio. Whether this ratio can more sensitively reflect the association between metabolic imbalance and breast cancer, and whether there are differences in gender or disease stages, remains to be further investigated. Therefore, we hypothesize that the UHR can serve as an integrated biomarker reflecting metabolic disorders and breast cancer odds. The purpose of this study is to explore the correlation between UHR and breast cancer odds, so as to fill the gap in existing research and provide new theoretical basis and practical directions for the early detection, odds stratification and metabolic intervention of breast cancer.

## Materials and methods

### Study population and data collection

**Figure 1:**
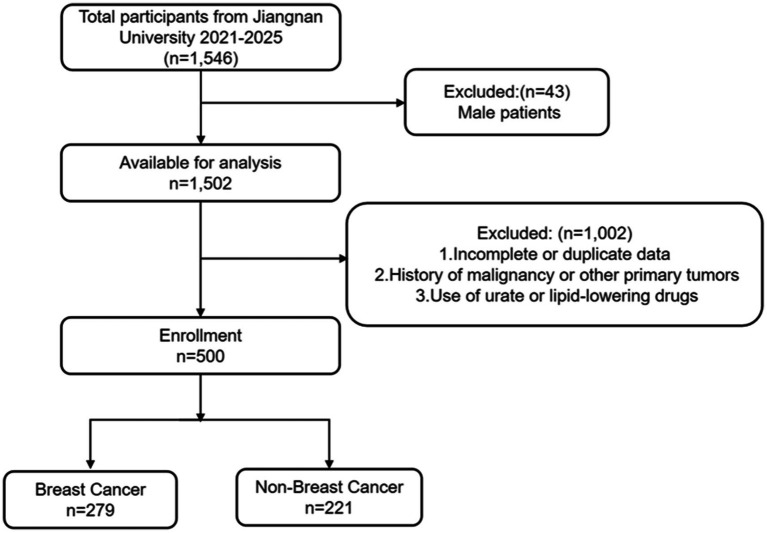
Flow chart of study subjects.

We retrospectively collected the clinical and pathological data of 500 female patients who visited the Affiliated Hospital of Jiangnan University from October 2021 to October 2025, including 279 patients with breast malignant tumors and 221 patients with benign breast nodules (Figure 1). The inclusion criteria were as follows: (1) All cancer patients were pathologically diagnosed with primary breast cancer; (2) Complete pretreatment laboratory data were available; (3) Complete clinicopathological and laboratory data were available. Exclusion criteria: (1) Male patients; (2) Incomplete information and duplicate data; (3) Patients with a history of malignant tumors or complicated with other primary tumors; (4) Patients who have used uric acid-lowering and lipid-lowering drugs. This study was granted ethical approval by the Medical Ethics Committee of the Affiliated Hospital of Jiangnan University (Approval No. JNMS01250043). The data of this retrospective study were anonymized, so the requirement for informed consent was waived.

### Data collection

We extracted demographic, clinical and pathological information for 500 individuals from the Affiliated Hospital of Jiangnan University’s database. All patients underwent serum biochemical examinations within one week before surgery, with complete data. The hospital used the Sysmex hematology analyzer for blood analysis and the Roche Cobas C702 biochemical analyzer to detect liver function and collect laboratory data including glucose, albumin, uric acid, cholesterol, triglycerides, high-density lipoprotein and low-density lipoprotein. Patients’ age, presence or absence of hypertension, diabetes mellitus, coronary heart disease, and smoking and drinking histories were collected from their medical records and pathological reports.

The calculation formula for laboratory data is as follows:

Uric Acid to High-Density Lipoprotein Cholesterol Ratio (UHR) = Serum Uric Acid (mg/dL) / High-Density Lipoprotein Cholesterol (mg/dL) * 100 (13).

### Statistical analysis

SPSS 26.0 statistical software was used for data processing and analysis in the statistical analysis. In this study, normally distributed continuous data are reported as Mean ± SD, and comparisons between two groups are made using the independent samples t-test. Skewed continuous data are reported as M (Q₁, Q₃) and compared using the Mann–Whitney U test. Categorical data are reported as n (%) and analyzed using the chi-square test or Fisher’s exact test; a *p* value of less than 0.05 is considered statistically significant for comparisons between groups. From the ROC curve, we derived the Youden index and the area under the curve (AUC) to quantify UHR’s diagnostic performance. Logistic regression was used to analyze the association between the UHR index and breast cancer, and three models were established for evaluation in the data analysis. Model 1 employed univariate logistic regression, whereas Model 2 incorporated multivariate logistic regression, accounting for covariates such as age, blood glucose, albumin, LDL, triglycerides, and total cholesterol. Model 3 extended Model 2 by further adjusting for hypertension, diabetes and coronary heart disease in a multivariate logistic framework. Statistical significance was set at a two-tailed *p* < 0.05.

## Results

### Baseline characteristics

**Figure 2:**
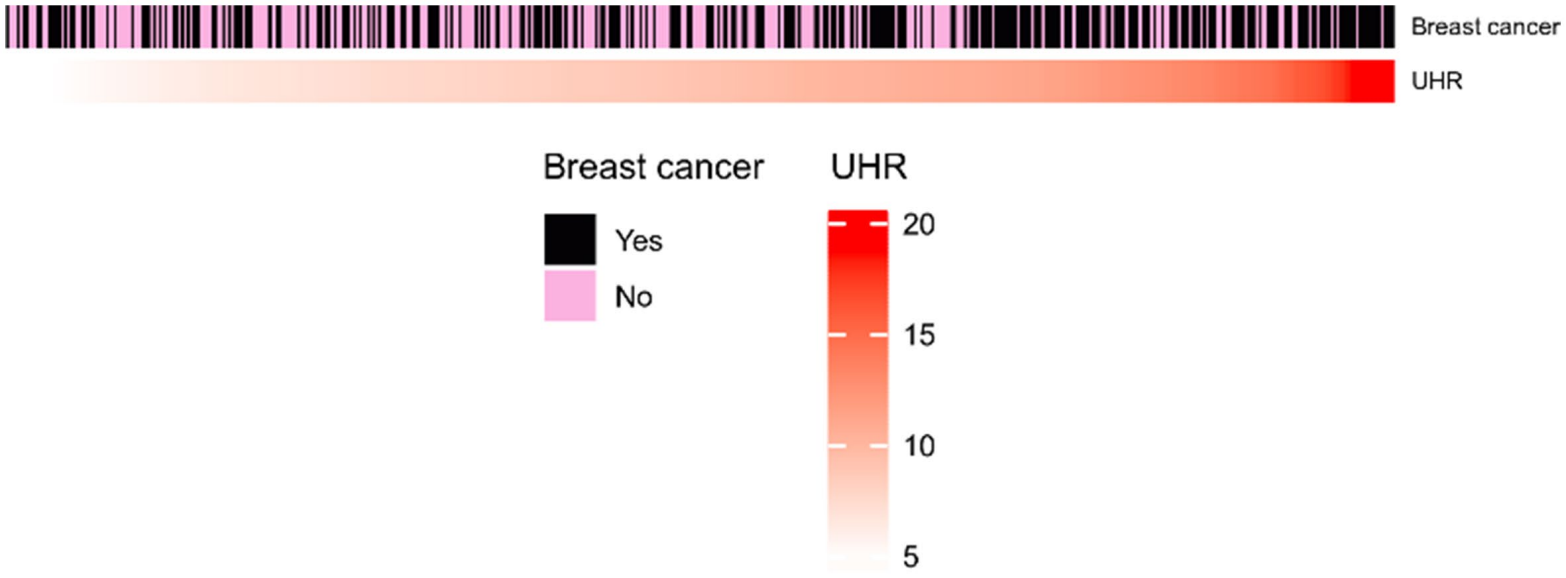
Heatmap of UHR index between breast nodules and breast cancer groups.

Table 1 presents the baseline characteristics of 500 patients in the Affiliated Hospital of Jiangnan University, where the demographic and clinicopathological characteristics of the patients as well as their preoperative laboratory test results are summarized. Figure 2 shows the distribution of the UHR index between the breast nodules and breast cancer groups.

**Table 1:** Clinical characteristics of the 500 patients.

Variables	Total (*n* = 500)	Non-breast cancer (*n* = 221)	Breast cancer (*n* = 279)	*P*
Age, M (Q₁, Q₃)	48.00 (36.00, 57.00)	37.00 (29.00, 48.00)	55.00 (46.50, 61.00)	**<0.001**
Glucose mmol/L, M (Q₁, Q₃)	4.94 (4.57, 5.37)	4.86 (4.57, 5.28)	5.00 (4.60, 5.46)	**0.028**
UA mg/dl, M (Q₁, Q₃)	4.72 (3.97, 5.58)	4.53 (3.85, 5.42)	4.79 (4.00, 5.68)	0.060
HDL-c mg/dl, M (Q₁, Q₃)	52.20 (44.47, 59.55)	54.91 (46.40, 61.10)	49.50 (42.54, 57.04)	**<0.001**
UHR, M (Q₁, Q₃)	9.09 (7.20, 11.33)	8.44 (6.78, 10.48)	9.91 (7.53, 12.17)	**<0.001**
Albumin g/dl, M (Q₁, Q₃)	4.12 (3.91, 4.45)	4.18 (3.94, 4.55)	4.08 (3.88, 4.34)	**0.001**
Tg mg/dl, M (Q₁, Q₃)	102.30 (69.97, 145.48)	82.37 (60.23, 116.03)	120.46 (85.91, 165.63)	**<0.001**
Tc mg/dl, M (Q₁, Q₃)	184.82 (164.23, 211.50)	183.66 (160.85, 206.47)	187.53 (166.45, 214.40)	0.172
LDL-c mg/dl, M (Q₁, Q₃)	115.80 (99.76, 135.33)	114.06 (98.21, 130.69)	117.15 (100.92, 139.97)	0.086
Hypertension, n (%)				**0.001**
No	431 (86.20)	203 (91.86)	228 (81.72)	
Yes	69 (13.80)	18 (8.14)	51 (18.28)	
Diabetes, *n* (%)				0.129
No	476 (95.20)	214 (96.83)	262 (93.91)	
Yes	24 (4.80)	7 (3.17)	17 (6.09)	

In this study, there were a total of 500 participants, of which 221 had breast nodules, making up 44.20%, and 279 had breast cancer, making up 55.80%. There were statistically significant differences between the two groups in age, glucose (mmol/L), HDL-c (mg/dl), UHR, albumin (g/dl), Tg (mg/dl), and hypertension (*p* < 0.05), and there were no statistically significant differences in UA (mg/dl), Tc (mg/dl), LDL-c (mg/dl), and diabetes (*p* > 0.05). The tumor group had a significantly older age, with an extremely significant difference, indicating that malignant tumor patients were mainly middle-aged and elderly, which was consistent with previous epidemiological studies (14). The levels of serum albumin and HDL-C were lower in the tumor group, indicating that hypoalbuminemia is an important marker of tumor-related malnutrition and chronic inflammation, and it is also consistent with the phenomenon of “tumor-related lipid metabolism reprogramming” (15). Figure 2 shows that most high UHR values belong to the breast cancer group.

### Association between UHR and breast cancer odds

Table 2 illustrates the association between the UHR index, dichotomized at the median, and the odds of breast cancer. In the three models, for each 100-unit increase in UHR, the odds of breast cancer increased by 9–13%, indicating that UHR is an independent odds factor with a robust association. Compared with the Q1 group (UHR < 0.091), the odds of breast cancer in the Q2 group (UHR ≥ 0.091) was significantly increased, with an OR of 1.63 (95% CI: 1.03–2.59, *p* = 0.038) in Model 3. That is, after comprehensive adjustment for confounding factors, the odds of breast cancer in patients with UHR in the Q2 group was 63% higher than that in the Q1 group. This suggests that UHR is independently and positively correlated with breast cancer odds in a dose-dependent manner, whether as a continuous or categorical variable, and has potential predictive value.

**Table 2:** UHR-breast cancer correlation.

UHR	Model 1 (OR95%CI)	*P*	Model 2 (OR95%CI)	*P*	Model 3 (OR95%CI)	*P*
Continues <0.091 ≥0.091	1.13(1.07–1.19) 1 (Reference) 2.09 (1.46–3.00)	<0.001 —— <0.001	1.09(1.02–1.17) 1 (Reference) 1.60 (1.01–2.53)	0.017 —— 0.046	1.10(1.02–1.18) 1 (Reference) 1.63 (1.03–2.59)	0.011 —— 0.038

Given that simultaneously testing three models might inflate Type I errors, Hochberg’s correction method was used to adjust the *p*-values in multiple comparisons. After correcting at the *α* = 0.05 level, the p-values for the three models (0.001, 0.038, 0.046) were all considered significant, which means there were no false positives.

### Diagnostic performance of UHR

**Figure 3:**
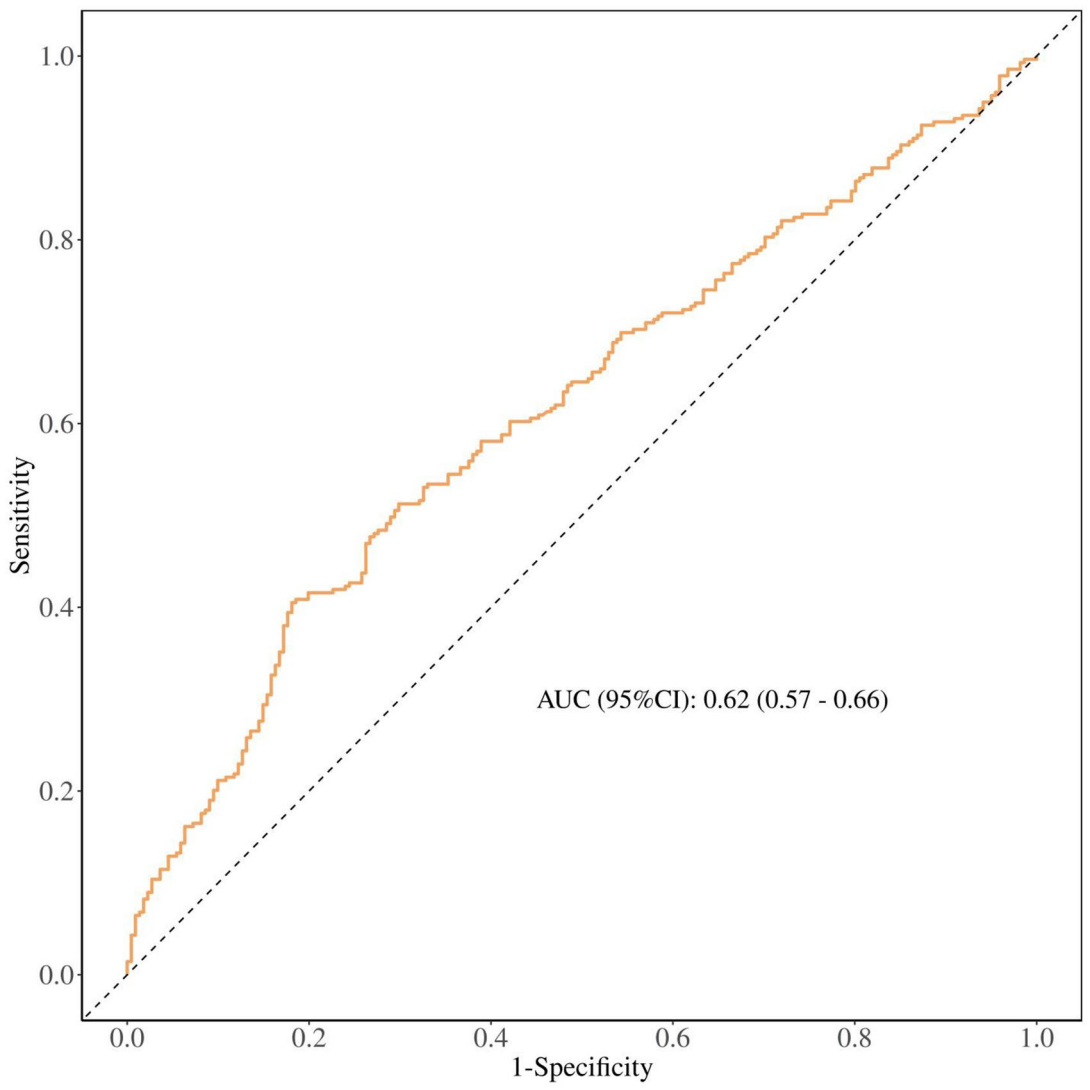
ROC curve analysis.

The ROC curve analysis (Figure 3) shows UHR’s accuracy in diagnosing breast cancer is 0.62 (95% CI 0.57–0.66). This means its overall diagnostic performance is just okay. So, future research should aim to boost its diagnostic performance through big cohort studies.

Table 3 shows how UHR performs in the diagnosis of breast cancer. Its sensitivity is 0.80 (0.75–0.85) and the negative predictive rate is 0.72 (0.65–0.79), showing it can fairly accurately identify people who actually have the disease, pointing to its potential value for initial screening in high-risk groups. However, with a specificity of just 41%, the false positive rate is pretty high, which means that this biomarker increases the number of misdiagnosed individuals. Furthermore, it still needs to improve in other areas.

**Table 3:** Performance metrics of the confusion matrix.

AUC (95%CI)	Accuracy (95%CI)	Sensitivity (95%CI)	Specificity (95%CI)	PPV (95%CI)	NPV (95%CI)	Cut off
0.62 (0.57–0.66)	0.58 (0.54–0.63)	0.80 (0.75–0.85)	0.41 (0.35–0.47)	0.52 (0.46–0.57)	0.72 (0.65–0.79)	0.107

PPV, positive predictive value; NPV, negative predictive value.

### Analysis of non-linear relationship

**Figure 4:**
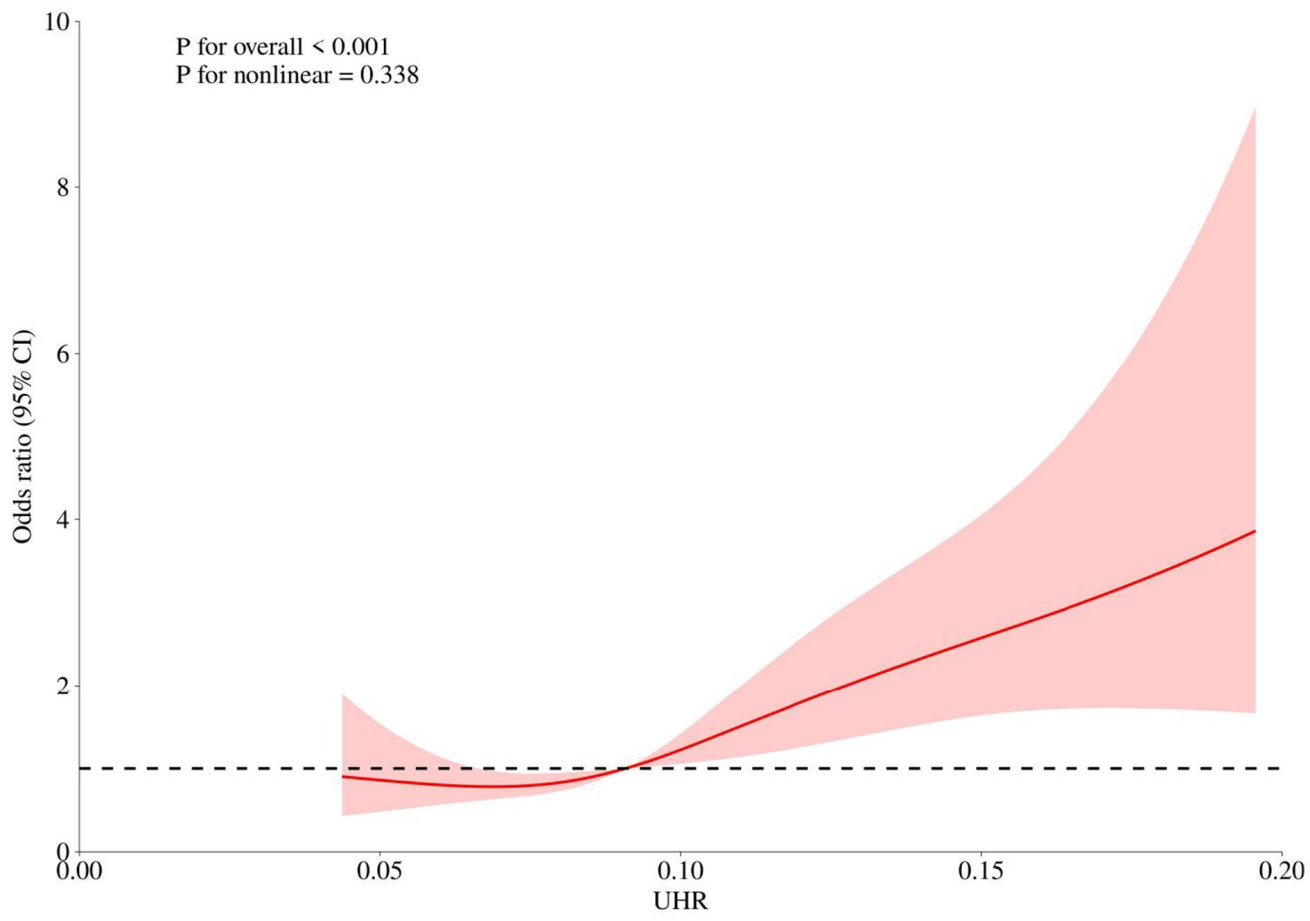
Restricted cubic spline analysis.

Restricted cubic spline (RCS) curves were used to explore the non-linear relationship between UHR and breast cancer odds. Restricted cubic spline regression analysis (Figure 4) showed a positive correlation between UHR and breast cancer odds (*P* for overall < 0.001, *P* for nonlinear = 0.338), indicating that the odds increases with the continuous elevation of UHR without a significant threshold effect.

## Discussion

Through a retrospective analysis, this study systematically explored the association between the serum uric acid to high-density lipoprotein ratio (UHR), a novel composite metabolic index, and the odds of breast cancer in Chinese women. Our main findings confirmed the initial hypothesis: the level of UHR was significantly elevated in breast cancer patients, and it was independently and positively correlated with breast cancer odds in a dose-dependent manner. This association remained robust after full adjustment for various confounding factors such as age, basic metabolic indicators and comorbidities. In addition, receiver operating characteristic (ROC) curve analysis indicated that UHR had moderate diagnostic efficacy, especially showing high sensitivity, which provided preliminary evidence for its use as an auxiliary screening tool.

The core value of this study lies in integrating two independent metabolic pathways, namely uric acid (UA) and high-density lipoprotein cholesterol (HDL-C), into a single indicator (15). On the other hand, HDL-C is known for its strong reverse cholesterol transport capacity, anti-inflammatory and antioxidant properties, and is regarded as “protective cholesterol.” A decrease in its level indicates an impairment of the body’s anti-tumor defense capacity (16, 17). Therefore, the elevation of UHR (i.e., relative increase in UA and/or relative decrease in HDL-C) accurately captures the dual imbalance state in the body where “tumor-promoting forces” are enhanced and “tumor-suppressive protection” is weakened. This may better reflect the overall metabolic disorder background related to tumor occurrence and development than any single indicator. Our findings are highly consistent with the “tumor metabolic reprogramming” theory, which states that cancer cells reshape their own and systemic metabolism to meet the energy and material needs of their rapid proliferation (18).

Our research results are both consistent with existing literature and represent an important advancement. Previous studies have separately reported that hyperuricemia is associated with an increased odds of various cancers (19, 20) as well as the association between decreased HDL-C levels after chemotherapy and poor prognosis in breast cancer patients (21, 22). However, these investigations primarily focused on linear or J-shaped associations of individual biomarkers. By introducing UHR, this study elevates the perspective from “isolated factors” to “network interaction.” We found a linear positive correlation between UHR and breast cancer odds, with no significant nonlinear relationship observed (P for nonlinear = 0.338), which provides a simpler basis for odds assessment in clinical practice. More importantly, in the multivariate model, UHR maintained independent predictive value, while the intergroup differences of certain single indicators (such as TC and LDL-C) were not significant. This suggests that as a composite indicator, UHR may have a stronger ability to identify signals among noise and can more sensitively reveal the metabolic oddss hidden in the population.

## Conclusion

In summary, this study is the first to reveal that the uric acid to high-density lipoprotein ratio (UHR) is an independent positive factor associated with the incidence of breast cancer. This research offers a new perspective on understanding the metabolic etiology of breast cancer. Future prospective studies are needed to confirm its clinical usefulness, dig deeper into the biological mechanisms, and ultimately offer new insights into metabolic prevention, as well as accurate diagnosis and treatment strategies for breast cancer.

## Limitations

Our limitations include the following: 1. The retrospective design cannot establish the causal sequence; 2. The single-center sample may have regional bias; 3. The lack of an external validation set means the model’s generalization ability remains to be confirmed.

## Future research directions

In the future, we can conduct prospective, multi-center cohort studies to verify the dose–response relationship between UHR and breast cancer incidence; carry out stratified studies by gender, menopausal status, and molecular subtypes to clarify the cut-off points and weights of UHR in different subgroups. We can also combine metabolomics and proteomics to clarify the core pathways through which UHR affects tumor proliferation and metastasis. Meanwhile, by designing lifestyle or drug intervention trials, we can evaluate whether reducing UHR can reduce the incidence of breast cancer or improve prognosis.

## Data Availability

The original contributions presented in the study are included in the article/supplementary material, further inquiries can be directed to the corresponding author/s.
